# Complete genome sequence of *Desulfovibrio* sp. strain TCA, a sulfate-reducing bacterium isolated from river sediment

**DOI:** 10.1128/mra.00202-25

**Published:** 2025-06-23

**Authors:** Tongyue Zhou, Huijuan Jin, Ke Shi, Yue Weng, Jingjing Wang, Yiru Cui, Jun Yan, Xiuying Li

**Affiliations:** 1 Key Laboratory of Pollution Ecology and Environmental Engineering, Institute of Applied Ecology, Chinese Academy of Sciences 74763 , Shenyang, Liaoning, China; 2 University of Chinese Academy of Sciences, Beijing, China; Rochester Institute of Technology, Rochester, New York, USA

**Keywords:** sulfate-reducing bacteria, amino acid metabolism, sulfate reduction, dechlorination

## Abstract

A new member affiliated with the genus *Desulfovibrio*, designated as strain TCA, was isolated from a dechlorinating enrichment culture originating from a freshwater river sediment. Its genome includes a 3.5 Mb chromosome with a G + C content of 66.2% and a 3.2 kb plasmid with a G + C content of 59.9%.

## ANNOUNCEMENT

A dechlorinating enrichment culture was established using sediment from the Xi River in Shenyang, Liaoning, China (41.7922°N, 123.4328°E). Following repeated transfers, a *Desulfovibrio* sp. isolate, designated strain TCA for its ability to reductively dechlorinate 1,1,1-trichloroethane (1,1,1-TCA), was obtained in 2023 by colony picking on an agar plate. PCR amplification of the 16S rRNA gene and Sanger sequencing were performed using the universal bacterial primers 27F and 1492R (1). Strain TCA was cultivated in bicarbonate-buffered (pH 7.2, 30 mM), reduced mineral salts medium supplemented with 20 mM lactate, 413 µmol hydrogen, and 5 mM sodium sulfate. Strain TCA shares 98.6% 16S rRNA gene identity with *Desulfovibrio aminophilus* DSM 12254 (GenBank accession number NR_024916), which lacks the ability to perform reductive dechlorination.

Cells were collected from 1.6 L strain TCA culture, and genomic DNA was extracted using the SDS method (2). Whole-genome sequencing was performed using a combination of Illumina NovaSeq PE150 and PacBio Sequel II platforms. For Illumina sequencing, the DNA was fragmented to ~350 bp, and libraries were constructed using the NEBNext Ultra DNA Library Prep Kit (New England Biolabs, Ipswich, MA, USA) following the manufacturer’s recommendations. The Illumina sequencing generated a total of 5,872,516 paired-end reads (2 х 150 bp) with a Q30% score of 93.21%. For PacBio long-read sequencing, the DNA sample underwent 10 kb BluePippin size selection (Sage Science, Beverly, MA, USA), and a library was prepared using the SMRTbell Template Prep kit 3.0 (Pacific Biosciences, Menlo Park, CA) following the manufacturer’s instructions (3). The PacBio sequencing generated a total of 160,141 long reads (N50 = 49,582 bp). Default settings were used for bioinformatics tools unless otherwise stated. Raw read quality control, error correction, and adapter trimming were performed using the SMRT Link v10.1. The quality of PacBio and Illumina data sets was evaluated using FastQC (4). Raw sequencing reads were assembled using Unicycler v0.50 (5).

The genome was assembled at 12× coverage, yielding a circular 3.5 Mb chromosome with a G + C content of 66.2% and a circular 3.2 kb plasmid with a G + C content of 59.9%. Annotation using the NCBI Prokaryotic Genome Annotation Pipeline v6.7 (6) identified 3,355 genes, including 3,296 coding sequences (CDSs), 2 rRNA operons (5S/16S/23S), 49 tRNAs, and four ncRNAs (6). Comparative genomic analysis using GGDC v3.0 (7) and JSpeciesWS v4.1.1 (8) revealed that strain TCA shares an 87.8% digital DNA-DNA hybridization value and 98.4% average nucleotide identity with *Desulfovibrio aminophilus* DSM 12254 (GenBank accession number GCA_000422565), supporting the classification of strain TCA within the genus *Desulfovibrio*. Strain TCA genome harbors genes involved in amino acid metabolism, including ornithine cyclodeaminase family protein (locus tag # ACEB74_00945). It also has all essential genes for the sulfate-reduction pathway, such as adenylyl-sulfate kinase (locus tag # ACEB74_03910), *aprA* (locus tag # ACEB74_14555), and dissimilatory sulfite reductase D family protein (locus tag # ACEB74_13760). Moreover, it has genes for hydrogen metabolism, including the ones coding for hydrogenase maturation protease (locus tag # ACEB74_01155) and hydrogenase maturation nickel metallochaperone (*hypA*; locus tag # ACEB74_07200).

## Data Availability

Strain TCA genome has been deposited in the GenBank under the accession numbers CP168306 (chromosome) and CP168307 (plasmid). The BioSample and BioProject numbers are SAMN43306170 and PRJNA1151236, respectively. The raw sequencing reads have been submitted to the Sequence Read Archive (SRA) under the accession numbers SRR32041244 (PacBio sequencing) and SRR32041245 (Illumina sequencing).
